# Validation of PREM-PS, a patient-reported experience instrument, in a randomized controlled trial of pregnant women undergoing prenatal screening

**DOI:** 10.1186/s41687-026-01020-5

**Published:** 2026-03-07

**Authors:** Alix Dubeau, Meryeme El Balqui, Denis Talbot, François Rousseau, Sylvie Langlois, Weihong Chen, François Audibert, Emmanuel Bujold, Jean-Claude Forest, Suélène Georgina Dofara, France Légaré

**Affiliations:** 1 Département de mathématiques et de statistique, Université Laval, Québec, QC Canada; 2 VITAM – Centre de recherche en santé durable, Centre intégré universitaire de santé et services sociaux (CIUSSS) de la Capitale- Nationale; Université Laval, Québec, QC Canada; 3 Département de médecine sociale et préventive, Faculté de médecine, Québec, Canada; 4 Axe santé des populations et pratiques optimales en santé, Centre de recherche du CHU de Québec – Université Laval, Québec, QC Canada; 5 Département de biologie moléculaire, de biochimie médicale et de pathologie, Faculté de médecine, Université Laval, Québec, QC Canada; 6 Department of Medical Genetics, Faculty of Medicine, University of British Columbia, Vancouver, BC Canada; 7 Centre de recherche du CHU Ste-Justine – Université de Montréal; Département de Gynécologie-Obstétrique, Université de Montréal, Montréal, QC Canada; 8 Axe Reproduction, santé de la mère et de l’enfant, Centre de recherche du CHU de Québec – Université Laval, Québec, QC Canada; 9 Département d’obstétrique et gynécologie, Faculté de médecine, Université Laval, Québec, QC Canada; 10 Département de médecine de famille et de médecine d’urgence, Faculté de médecine, Université Laval, Québec, QC Canada

**Keywords:** Prenatal screening, Patient-reported experience measures (PREMs), Psychometric validation, Structural validity, Cross-cultural validity, Internal consistency, Factor analysis, First-tier cfDNA prenatal screening

## Abstract

**Background:**

Patient-reported experience measures are valuable instruments for assessing care quality in alignment with the Triple Aim framework. We sought to validate the PREM-PS questionnaire (Patient Reported Experience Measure – Prenatal Screening) by evaluating its psychometric properties (structural validity, internal consistency, and cross-cultural validity) for Canadian French-and English-speaking populations.

**Methodology:**

This secondary analysis used data from a prospective, open-label, multicenter randomized trial in Quebec and British Columbia (2019–2023). Pregnant women aged 19+ were randomized 2:1 to receive either first-tier cell-free DNA (cfDNA) or traditional biochemical screening for chromosomal anomalies (T21, T18, T13). Participants completed the PREM-PS, a 10-item questionnaire on a 5-point Likert scale, at 22 weeks of pregnancy. Exploratory factor analysis, conducted separately for French and English questionnaires, used half the data to identify underlying factors. Confirmatory factor analysis of the remaining data evaluated structural validity. Internal consistency was assessed using Cronbach’s alpha, and cross-cultural validity through measurement invariance analyses. Mean scores were compared across arms using linear mixed models.

**Results:**

Of 7815 enrolled pregnant women, 3398 completed the French questionnaire and 2875 the English version (80.3% response rate, 96.4% completion rate). Mean age was 32.11 years (SD = 4.01), with 65.1% identifying as White. Factor analyses retained seven items grouped into two latent factors interpreted as Communication and Professionalism. Standardized loadings from exploratory factor analysis ranged from 0.66–0.93 (French) and 0.69–0.96 (English), while standardized parameter estimates from the confirmatory factor analysis ranged from 0.67–0.98 (French) and 0.62–0.98 (English). Cronbach’s alpha exceeded 0.85 for both factors in French and 0.88 in English. No significant differences were observed between the two study arms for either Professionalism or Communication scores, indicating comparable patient-reported experiences regardless of screening method.

**Conclusion:**

The PREM-PS demonstrates adequate validity for assessing patient-reported experiences of pregnant women undergoing prenatal screening in both Canadian French and English, with potential to guide improvements in prenatal care.

**Trial registration:**

ClinicalTrials.gov, NCT03831256. Registered February 5^th^, 2019, https://clinicaltrials.gov/study/NCT03831256?term=NCT03831256&rank=1

## Abstract (plain language summary)

We developed and validated the first French and English questionnaires specifically designed to measure pregnant women’s experiences during prenatal screening. The questionnaire measures two core physician skills from Canada’s CanMEDS framework: Communication (how well providers listen and enable patient-centered dialogue) and Professionalism (clinical competence). Our study found that women receiving newer genetic screening methods (cfDNA) and traditional biochemical screening reported similar experiences, suggesting that the type of screening does not significantly impact how patients perceive their quality of care.

## Background

Pregnant women face many difficult decisions along their trajectory of prenatal care [1]. One of the most difficult decisions during pregnancy is whether to undergo prenatal screening for conditions such as fetal aneuploidy, knowing it may lead to further, increasingly complex choices—including invasive diagnostic testing, or deciding between pregnancy termination and preparing for a child with special needs [1]. In recent years, cell-free DNA (cfDNA) prenatal screening (commonly referred to as Non-Invasive Prenatal Testing, or NIPT) using maternal blood has been added to the various technical approaches to screening for fetal aneuploidy [2]. According to a systematic review [3], cfDNA screening offers higher sensitivity and specificity for trisomies 21, 18, and 13 than traditional prenatal screening methods. Indeed, due to its high sensitivity, after an initial high-risk serum screening, if a second-tier cfDNA screening indicates there is a low risk of fetal aneuploidy, there is no need for invasive confirmatory tests such as amniocentesis, which carry a risk of pregnancy loss. Additionally, cfDNA screening provides results earlier and faster when used as a first-line test compared to standard methods [4]. In Canada, at the time of this study, cfDNA screening was offered by the governments of several provinces as a second-line screening test.

Although the clinical effectiveness of cfDNA screening is well-established [3], there is limited understanding of how pregnant women experience prenatal screening—an aspect of healthcare aligned with the Triple Aim framework, which seeks to improve care experiences, enhance population health, and reduce healthcare costs [5]. Indeed, the use of patient-reported experience measures (PREMs) is increasingly recognized internationally as a way to assess healthcare quality and patient safety [6] as a complement to the assessment of patient outcomes and efficiency [5]. PREMs focus on the measurable aspects of the care process, such as interactions with healthcare providers, rather than the outcomes of care itself [7]. The aim of validating a PREM specifically for prenatal screening is to capture pregnant women’s experiences in a standardized and psychometrically sound manner, and, ultimately, to provide insight on how this experience can be improved, for example through continuing professional development for healthcare providers.

To the best of our knowledge, no studies to date have documented or validated the psychometric soundness of a PREM questionnaire specifically focused on the prenatal screening experience, a gap which highlights gender inequities in studies of patient-centered experiences of health care [8]. A study in Norway did describe the development and evaluation of the Norwegian Pregnancy and Maternity care Patient Experience Questionnaire (PreMaPEQ), a broader instrument measuring women’s experiences across pregnancy, birth, and postnatal care [9]. This questionnaire included key interactions related to prenatal screening—such as discussions about screening, openness to questions, and the perceived competence of healthcare providers. However, it extended beyond the context of prenatal screening and was not validated for use among culturally diverse populations. Canada is an officially bilingual country, with 22% of its population speaking French as their first language. Many Canadian immigrants are also French speakers. For example, in Quebec, where 84% of French speakers live, the largest proportion of immigrants come from France, followed by other French-speaking countries such as Haiti and Algeria [10]. Therefore, we sought to validate a patient-reported experience measure specific to prenatal screening—the PREM-PS questionnaire—and to evaluate its structural validity, internal consistency, and cross-cultural validity in both French and English-speaking populations.

## Methods

### Study design

We performed a secondary analysis, using data from a prospective, open-label, multicenter 2:1 randomized clinical trial conducted from 2019 to 2023. We reported our analysis according to the COSMIN guidelines for studies on measurement properties of patient reported outcome measures [11]. The trial was registered at ClinicalTrials.gov (ID: NCT03831256). The trial began only after approval from the Ethics Review Committees of both The University of British Columbia (UBC) and the CHU de Québec – Université Laval. (UBC Research Ethics Board under #H18-02062 and Comité d’éthique du CHU de Québec #MP-20–2019–4332).

### Population

Eligible participants included pregnant women aged 19 years or older who consented to prenatal screening between 10 and 14 weeks of gestation as determined by ultrasound. Exclusion criteria included known fetal anomalies at recruitment, multiple pregnancies, prior twin death, planned procedures such as chorionic villus sampling or amniocentesis for a known genetic condition, women over 40 years old in Quebec (who already qualify for public first-tier cfDNA screening and therefore were not eligible for randomization), those with cancer or a history of cancer, blood transfusions within the last month, or prior stem cell or organ transplants.

### Intervention

#### Intervention arm

The only prenatal blood screening test for fetal aneuploidy offered in the intervention arm was universal cell-free DNA (cfDNA) prenatal screening. Pregnant women between the 10th and 14th weeks of pregnancy were invited to provide a 30 ml blood sample. Split into two tubes, 20 ml of the sample was subjected to clinical cfDNA screening targeting trisomies 21, 18, and 13, while the other 10 ml was saved for further research purposes such as developing new tests. The cfDNA screening results were available within 7 to 10 days. If they indicated a higher probability of a fetal chromosomal anomaly, an invasive diagnostic test (e.g., amniocentesis) was offered to the woman, followed by chromosomal analysis.

#### Control/standard care arm

Pregnant women in the standard care arm underwent standard biochemical screening involving two blood tests with or without nuchal translucency, the first test between the 10th and 14th week of gestation and the second between the 14th and 16th week (although accepted up to 20 weeks). If screening results were above screen cut-offs, the woman was offered either second tier cfDNA screening (for T21, T18, T13) or an invasive diagnostic test consisting of either chorionic villi sampling or amniocentesis depending on the gestational age. If the cfDNA screening result in the standard care arm was positive (high-risk), the woman was offered a diagnostic test followed by chromosomal analysis. If the result was negative (low-risk), no further testing was performed. In both study arms, ultrasound scans were offered in keeping with the standard clinical care.

### Data collection

The PreMaPEQ questionnaire was originally designed to capture women’s experiences with pregnancy, childbirth, and postnatal care [9]. It is a publicly available instrument published without restrictions on adaptation. We reduced and modified it to address the specific context of prenatal screening and translated it into French. First, items from PreMaPEQ that were relevant to prenatal screening were selected to create an initial English version of the PREM-PS. Cognitive debriefing was conducted among 5 lay persons to review the clarity of the wording. The English version was then translated into French and back-translated into English by two independent non-scientific translators to ensure linguistic accuracy and accessibility for the Canadian context [12]. Cognitive interviewing was then conducted with 12 pregnant women to assess the comprehensibility, cognitive equivalence, and cultural relevance of the French version, following established guidelines for translation and cultural adaptation of patient reported outcome measures [13]. Through semi-structured interviews and a questionnaire, participants were asked about item clarity (e.g., “Were some sentences difficult to understand?”), appropriateness (e.g., “Were some sentences inappropriate or offensive?”), and completeness (e.g., “Is there anything else that could have been included?”). This process led to modification of the French response scale and refinement of one item to avoid redundancy. The final 10 items focused on key interactions during prenatal care such as discussions about screening and the healthcare provider’s openness to patient questions (Table 2). Each item was rated on a 5-point Likert scale: Strongly disagree (1), Disagree (2), Neither agree nor disagree (3), Agree (4), Strongly agree (5). Details of item sources and modifications are provided in Appendix 1.

Data collection occurred at the time of recruitment for the study, during which socio-demographic information such as age, province, and ethnicity was recorded. At 22 weeks, all participants were invited to complete the self-administered PREM-PS questionnaire online using the secure REDCap platform [14].

### Sample size and randomization

Based on Everitt’s recommendations [15], a minimum participant-to-item ratio of 10:1 is required to minimize sampling errors and ensure robust psychometric analyses. Our study exceeded this threshold, as the questionnaire contained 10 items and the data included 4313 responses for the French version and 3502 for the English version. A post-hoc power analysis for the comparison of PREMs between randomization arms revealed a power of 96% to detect small effect sizes of 0.1 and 0.2, and 99% to detect large effect sizes of 0.8.

Randomization for the original trial from which our data are extracted was conducted in variable block sizes and dynamically stratified by clinical recruitment site and age (younger than 35 years or 35 years and older), given the increased likelihood of trisomy 21 with maternal age [16]. Dynamic stratification ensured proportional assignment to treatment groups based on the characteristics of the first randomized participant in each block, reducing bias and enabling balanced comparisons. Randomization was managed by an independent center.

### Statistical analysis

Descriptive analyses were initially conducted to summarize the demographic and clinical characteristics of the sample. Additionally, characteristics of participants with missing data were examined to identify any potential patterns or biases in the missing responses. Descriptive statistics for single items including mean, median, standard deviation, minimum and maximum were calculated.

We first sought to determine if the items measured a few common factors (i.e. components of the patient experience) using factor analyses. To first assess whether the data were suitable for factor analyses, a Kaiser-Meyer-Olkin (KMO) statistic was calculated, with values above 0.80 considered appropriate [17], and Bartlett’s test of sphericity was calculated to confirm that the data were suitable for factor analysis [18]. Because we had no assumption on the number of factors that the items measured, we first performed an exploratory factor analysis (EFA) separately for the French and English versions of the questionnaire, using a random half of each dataset to explore the underlying factor structure. EFA was performed using the Full Information Maximum Likelihood estimator, which produces valid estimates under the assumption that data are missing at random. The Oblimin factor rotation was selected based on the presumed dependence of factors. Items were assigned to the factor with the highest loading provided that the loading exceeded 0.5. We aimed to retain a consistent model structure for both French and English versions.

Internal consistency was assessed using Cronbach’s alpha to determine how closely the items were related as a group for each individual identified factor of the PREM-PS questionnaire (measuring the reliability of the identified factors), with values > 0.7 indicating acceptable internal consistency and values > 0.95 suggesting potential redundancy [19]. Corrected item-total correlations [20] were calculated to assess the alignment of individual items with their respective factors, providing insight into the scale’s coherence and identifying potentially problematic or misaligned items. A threshold of 0.5 was set to indicate adequate correlation, ensuring that all retained items contributed meaningfully to their respective factors. Factor correlations were examined. Strong correlations (>0.80 or >0.85) were considered indicative of low discriminant validity, potentially warranting a more parsimonious factor structure [21]. Confirmatory factor analysis (CFA) was then conducted jointly on the random half of the data not used for the EFA in both languages using the Weighted Least Squares Mean and Variance-adjusted (WLSMV) estimator to assess structural validity, which refers to the extent to which questionnaire scores accurately reflect the underlying factors of the construct being measured, and cross-cultural validity. WLSMV respects the ordinal nature of Likert scale data and provides robust parameter estimates without assuming multivariate normality [22]. Structural validity was determined based on the model fit indices, including the Comparative Fit Index (CFI) and the Root Mean Square Error of Approximation (RMSEA). Acceptable fit was indicated by CFI values above 0.90 and RMSEA values below 0.08, providing an indication of model adequacy [23, 24]. Additionally, modification indices (MIs) were examined to assess potential links between items and factors for model improvement. MIs greater than 3.84 [25] were investigated and considered for inclusion if they were conceptually meaningful. Cross-cultural validity was determined by testing for measurement invariance across language groups following a sequential model comparison approach, where increasingly restrictive constraints were applied. First, a configural model was estimated, imposing only the same factorial structure across language groups. Then, a weak invariance model constrained factor loadings to be equal between language groups, followed by a strong invariance model that further constrained intercepts. Likelihood ratio tests were used for model comparisons.

Once an appropriate factorial solution was established, factor scores were calculated using the factor loadings and the correlations between factors. The regression method, commonly employed for estimating factor scores, was applied in this study. First, the measured items were standardized into z-scores. These standardized scores were then multiplied by the inverse of the bivariate correlation matrix of the indicators and the matrix of factor loadings [26]. Linear mixed models were used to compare PREM-PS scores across the two randomized arms (first-tier cfDNA prenatal screening versus standard care), adjusting for potential confounders such as age and a random effect for the clinical recruitment site. To address potential bias related to non-response to the PREM-PS questionnaire, inverse probability of censoring weighting was applied. A logistic regression model was used to estimate the probability of questionnaire completion for each participant, with non-response as the dependent variable. Independent variables included socio-demographic and clinical characteristics such as age, ethnicity, and recruitment site. The inverse of these probabilities were then used as weights in the linear mixed-effects models. Participants with a higher likelihood of non-response were assigned greater weights to ensure that their characteristics were proportionally represented in the analysis. This approach accounts for individuals with similar characteristics who did not complete the questionnaire, thereby reducing the risk of bias due to non-response. To obtain confidence intervals that accounted for the entirety of the analysis, non-parametric bootstrap resampling was employed. Using the percentile method, 1000 bootstrap samples were generated by randomly resampling participants with replacement. Confidence intervals were derived directly from the distribution of the bootstrap estimates. All analyses were conducted in R [27] version 4.3.1 and the *lavaan* [28] package version 0.6–18 for factorial analysis.

Finally, the factorial solution was compared with the CanMEDS physician competency framework to assess conceptual alignment between the PREM-PS and established medical competencies.

## Results

### Flow and characteristics of participants

**Fig. 1:**
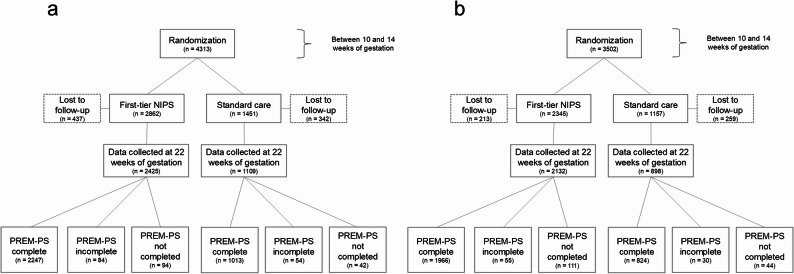
Participant flowchart by language of the PREM-PS questionnaire (**a**: French, **b**: English)

In the French sample, 78.8% of the initial participants (4313) responded to the PREM questionnaire, resulting in 3398 surveys. Among these, 3260 (95.9%) were fully completed while 138 (4.1%) had incomplete responses. Similarly, in the English sample, 82.1% of the initial participants (3502) responded, providing 2875 surveys. Of these, 2790 were fully completed (97.0%) and 85 (3.0%) were incomplete. The overall response rate across both language groups was 80.3%, with an average completion rate of 96.4%. Figure 1 illustrates the flowchart of participants by randomization arm and language of the PREM-PS questionnaire.

#### Descriptive statistics

A total of 6049 pregnant women were included in this analysis, all of whom provided a fully or partially completed questionnaire along with socio-demographic information. The mean age was 32.11 years (SD = 4.01). The majority of pregnant women identified as White (65.1%), followed by East Asian (11.9%). Other ethnicities, including Arab, South Asian, and South East Asian, each accounted for less than 5% of the population. Clinical characteristics showed low overall prevalence of diabetes (0.9%), hypertension (1.1%), smoking (2.2%) and pre-pregnancy pre-eclampsia (2.3%). Detailed characteristics by randomization arm are presented in Table 1.

**Table 1:** Descriptive statistics of participants by randomized arm

Characteristics	Overall *n* = 6049	Standard care *n* = 1837	cfDNA prenatal screening *n* = 4212
Mean (SD) age (years)	32.11 (4.01)	32.08 (3.99)	32.15 (4.02)
Race/Ethnicity, n (%)			
Arab (Egypt, Iraq, Jordan, Lebanon)	139 (2.3)	42 (2.3)	97 (2.3)
East Asian (China, Japan, Korea, Taiwan)	711 (11.9)	204 (11.1)	507 (12.0)
West Asian (Turkey, Iran, Afghanistan)	48 (0.8)	14 (0.8)	34 (0.8)
South East Asian (Malaysia, Indonesia, Vietnam, Cambodia)	195 (3.2)	49 (2.7)	157 (3.7)
South Asian (Indian, Sri Lanka, Pakistan, Bangladesh)	206 (3.4)	45 (2.4)	150 (3.6)
Aboriginal (First Nations, Métis, Inuit)	9 (0.1)	3 (0.2)	6 (0.1)
White (European descent)	3939 (65.1)	1247 (67.9)	2692 (63.9)
Jewish	6 (0.1)	102 (5.6)	245 (5.8)
Latin American/Hispanic	238 (3.9)	2 (0.1)	4 (0.1)
Black (African descent)	112 (1.9)	71 (3.9)	167 (4.0)
Black (Caribbean descent)	99 (1.6)	30 (1.6)	82 (1.9)
Mixed ethnicity or other	347 (5.7)	28 (1.5)	71 (1.7)
Diabetes = Yes (%)	53 (0.9)	13 (0.7)	40 (0.9)
Hypertension = Yes (%)	64 (1.1)	18 (1.0)	46 (1.1)
Smoking = Yes (%)	135 (2.2)	49 (2.7)	86 (2.0)
Previous History of Pregnancy Preeclampsia = Yes (%)	137 (2.3)	44 (2.4)	93 (2.3)

#### Loss to follow up

Ethnicity was the sociodemographic characteristic that most distinguished participants lost to follow-up or those who partially completed the questionnaire from those who fully completed it. Among participants who responded to the francophone questionnaire, White participants (European descent) accounted for 81.5% of those who fully completed the questionnaire, compared to only 57.4% of participants who did not complete or only partially completed it. For participants who responded to the anglophone questionnaire, White participants represented 45.9% of those who fully completed the questionnaire, compared to 33.8% of those who did not complete or partially completed it. Characteristics of pregnant women by the language of completion of the PREM-PS questionnaire are provided in Appendix 2.

### Descriptive statistics of items

The descriptive statistics for each item of the PREM-PS are presented in Table 2, stratified by the French and English versions of the questionnaire. Across both language groups, the highest mean scores were observed for items related to politeness and respect as well as to openness to questions among healthcare professionals, specifically Item 1 (“I was treated with politeness and respect by my health professional”) and Item 3 (“I felt that the health professional was open to questions during prenatal visits.”) These items had mean scores of 4.79 and 4.62 in the French questionnaire and 4.53 and 4.46 in the English questionnaire, respectively. The lowest mean scores were observed for Item 7 (“I think the health professional influenced my decision to have screening or to not have screening”) with means of 3.08 (French) and 3.02 (English). Additionally, higher variability was noted for Item 7 between languages.

**Table 2:** Descriptive statistics for each PREM-PS item, stratified by language

Item		French PREM-PS Questionnaire							English PREM-PS Questionnaire						
		N	Mean	Median	SD	Min	Max	Extreme responses (% Min | % Max)^a^	N	Mean	Median	SD	Min	Max	Extreme responses (% Min | % Max)^a^
1	I was treated with politeness and respect by my health professional.	3388	4.79	5	0.49	1	5	0.09 | 81.75	2870	4.53	5	0.87	1	5	3.51 | 66.78
2	I felt that the health professional spent enough time at the visits.	3388	4.38	5	0.89	1	5	1.09 | 57.74	2865	4.22	4	0.98	1	5	2.54 | 48.17
3	I felt that the health professional was open to questions during prenatal visits.	3388	4.62	5	0.65	1	5	0.21 | 69.48	2870	4.46	5	0.82	1	5	1.84 | 59.72
4	I felt that the health professional cared about me.	3388	4.49	5	0.79	1	5	1.03 | 62.36	2866	4.35	5	0.85	1	5	1.95 | 52.38
5	I felt that the health professional spent enough time discussing prenatal screening with me.	3344	4.34	5	0.86	1	5	0.74 | 52.83	2838	4.00	4	1.02	1	5	3.20 | 35.13
6	I felt that the health professional was open to questions about prenatal screening.	3342	4.52	5	0.67	1	5	0.03 | 60.12	2837	4.24	4	0.85	1	5	1.84 | 42.99
7	I think the health professional influenced my decision to have screening or to not have screening.	3341	3.08	3	1.34	1	5	17.98 | 19.01	2837	3.02	3	1.15	1	5	10.92 | 11.23
8	I trust my health professional in matters related to prenatal screening.	3345	4.58	5	0.61	1	5	0.03 | 62.63	2841	4.25	4	0.81	1	5	1.57 | 40.66
9	The health professional spent enough time discussing the results of my prenatal screening with me.	3338	3.85	4	1.13	1	5	4.12 | 34.87	2840	3.59	4	1.11	1	5	5.11 | 20.45
10	My experience with prenatal screening was what I expected based on the information I received prior to screening.	3337	4.12	4	0.9	1	5	1.32 | 38.91	2837	3.94	4	0.87	1	5	1.74 | 24.59

Note. SD: Standard deviation; ᵃ Percentage of respondents selecting the minimum (floor) or maximum (ceiling)

### Psychometric properties: structural validity, cross-cultural validity, and internal consistency

The overall KMO score was 0.88 for the French questionnaire and 0.91 for the English questionnaire, indicating strong sample adequacy for factor analysis. Additionally, Bartlett’s test of sphericity was statistically significant in both languages (*p* < 0.05), confirming that the data were suitable for factor analysis. Using EFA with Oblimin rotation, item 7 (“I think the health professional influenced my decision to have screening or to not have screening”) was removed due to low factor loadings of 0.244 in French and 0.365 in English, both below the 0.5 threshold. A two-factor model was almost identical in French and English. In both the French and English versions, factor 1 included items 1, 2, 3 and 4. In the French version, Factor 2 included items 5, 6, and 8, whereas the English version included items 5, 6, 8, 9, and 10. Standardized weights of the EFA model are presented in Table 3.

**Table 3:** Standardized weights of the exploratory two-factor model using Oblimin rotated factors, stratified by language

Item		French PREM-PS questionnaire		English PREM-PS questionnaire	
		Factor 1	Factor 2	Factor 1	Factor 2
1	I was treated with politeness and respect by my health professional.	0.670*	0.000	0.687*	−0.010
2	I felt that the health professional spent enough time at the visits.	0.700*	0.102*	0.824*	0.060*
3	I felt that the health professional was open to questions during prenatal visits.	0.925*	−0.063*	0.956*	−0.045*
4	I felt that the health professional cared about me.	0.783*	0.058*	0.865*	0.042
5	I felt that the health professional spent enough time discussing prenatal screening with me.	−0.071*	0.899*	−0.021	0.906*
6	I felt that the health professional was open to questions about prenatal screening.	0.037	0.850*	0.112*	0.800*
7	I think the health professional influenced my decision to have screening or to not have screening.	−0.084	0.244*	−0.101	0.365*
8	I trust my health professional in matters related to prenatal screening.	0.086*	0.662*	0.101*	0.740*
9	The health professional spent enough time discussing the results of my prenatal screening with me.	0.181*	0.436*	−0.076*	0.744*
10	My experience with prenatal screening was what I expected based on the information I received prior to screening.	0.044	0.473*	−0.080*	0.694*

Note. * = significant at 1% level

To favor a common model across both languages, the French two-factor structure was tested on the English data with CFA. This structure was selected to include only items meeting the 0.5 factor loading threshold in both languages; items 9 and 10 did not meet this criterion in French (loadings of 0.436 and 0.473), precluding their inclusion in a unified bilingual model. A summary of items retained and removed during the validation process is presented in Table 4. Before conducting the CFA, corrected item-total correlations were calculated using the two-factor model on the combined French and English data, and all correlations exceeded 0.5, with none approaching the threshold for concern (Table 5), indicating strong correlations between each item and its respective factor. Internal consistency (Cronbach’s alpha) for the two-factor model exceeded the a priori threshold of 0.7, with values of 0.85 for both Factor 1 and Factor 2 for the French questionnaire, and 0.90 and 0.88 for the English questionnaire (Table 6). For the CFA, Question 6 was recoded by collapsing the two lowest response categories due to an empty category in the French language group, and the WLSMV estimator’s use of listwise deletion for missing data resulted in the exclusion of 73 participants (2.3%): 45 French-speaking (2.6%) and 28 English-speaking (1.9%). CFA model comparisons using likelihood ratio tests revealed that weak invariance and strong invariance were not supported, leading to the retention of the configural model as the best-fitting solution. This indicates that while the factor structure is consistent across language groups, direct comparisons of mean scores between French and English versions should be interpreted with caution.

**Table 4:** Summary of items retained and removed during the validation process

Item	Status	Rationale
1–6, 8	Retained	Factor loadings ≥0.5 in both languages
7	Removed	Factor loadings < 0.5 (French: 0.244; English: 0.365)
9	Removed	Factor loading < 0.5 in French (0.436)
10	Removed	Factor loading < 0.5 in French (0.473)

**Table 5:** Corrected item-total correlations based on the two-factor model for the combined French and English data

Item		Factor 1	Factor 2
1	I was treated with politeness and respect by my health professional.	0.66	
2	I felt that the health professional spent enough time at the visits.	0.76	
3	I felt that the health professional was open to questions during prenatal visits.	0.85	
4	I felt that the health professional cared about me.	0.80	
5	I felt that the health professional spent enough time discussing prenatal screening with me.		0.83
6	I felt that the health professional was open to questions about prenatal screening.		0.88
8	I trust my health professional in matters related to prenatal screening.		0.70

**Table 6:** Cronbach’s alpha of the two-factor model by language of the PREM-PS questionnaire (French and English)

	French PREM-PS questionnaire	English PREM-PS questionnaire
Factor 1	0.85	0.90
Factor 2	0.85	0.88

**Fig. 2:**
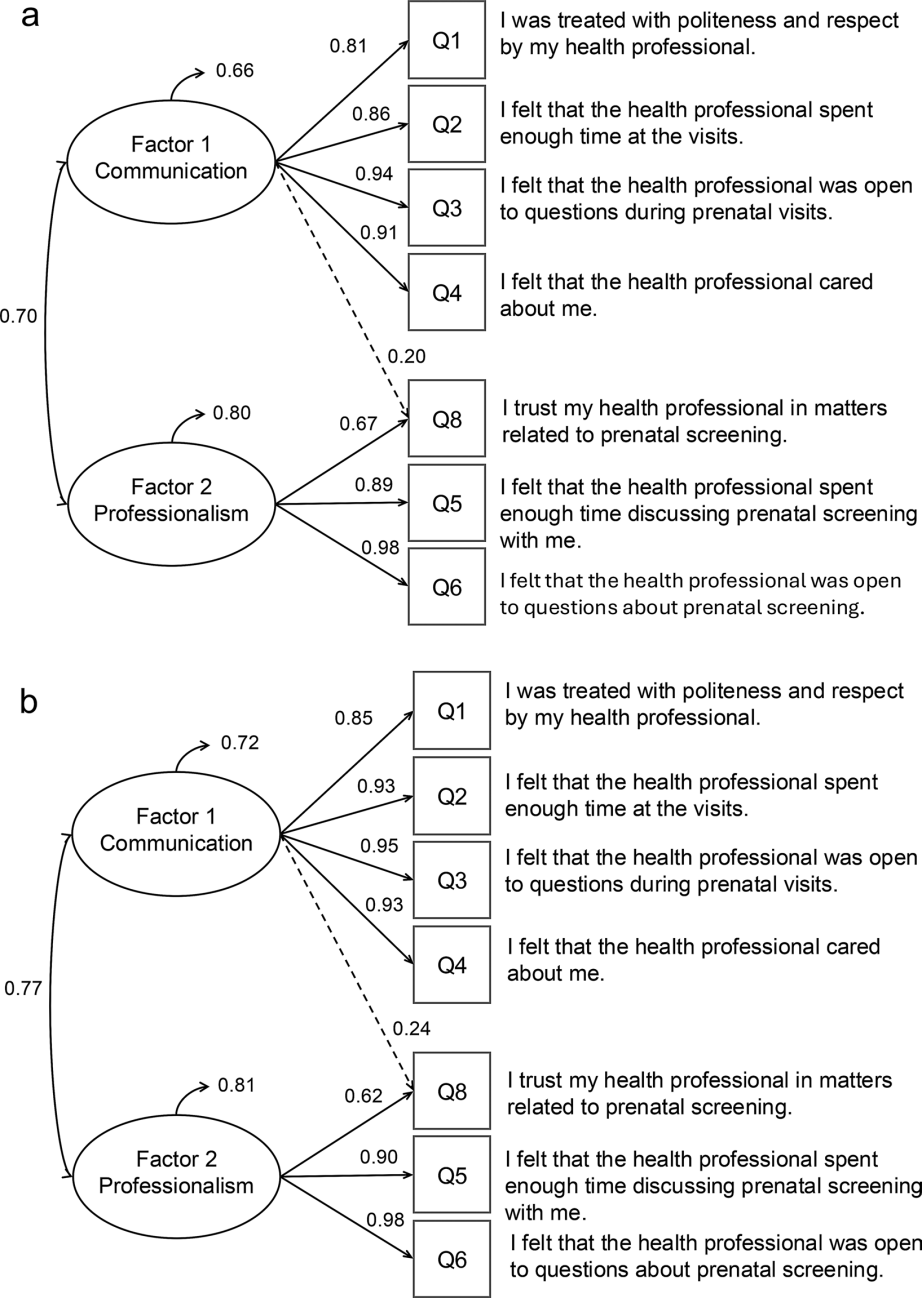
Standardized factor loadings, latent variable correlations, and factor variances from CFA by language (**a**: French, **b**: English)

The fit indices of the CFA model were strong. The CFI was 1.00, exceeding the threshold of 0.95. The RMSEA improved from 0.045 to 0.009 following the addition of a cross-loading between Question 8 and Factor 1. This modification was guided by a high modification index (MI = 42.66), indicating a significant potential improvement in model fit. The chi-square test was non-significant (*p* = 0.298), indicating good model fit to the observed data. Final results of the CFA model are presented in Fig. 2. Items 1, 2, 3, and 4 were grouped into Factor 1, while items 5, 6, and 8 were grouped into Factor 2, with an added link between item 8 and Factor 1. Correlation between factors was 0.70 in the French version and 0.77 in the English version, below 0.85, indicating that the factors were distinct but moderately related. Factor variances differed between language groups, with values of 0.66 for Factor 1 and 0.80 for Factor 2 in the French version, compared to 0.72 for Factor 1 and 0.81 for Factor 2 in the English version. These small differences suggest relatively consistent response patterns across language groups. Parameter estimates of CFA are presented in Table 7.

**Table 7:** Parameter estimates from CFA using the WLSMV estimator, by language of the PREM-PS questionnaire (French and English)

Latent Factor	Item		French PREM-PS questionnaire					English PREM-PS questionnaire				
			B	SE	Z	β	p	B	SE	Z	β	p
F1	1	I was treated with politeness and respect by my health professional.	1.000			0.813		1.000			0.851	
F1	2	I felt that the health professional spent enough time at the visits.	1.055	0.022	47.385	0.858	***	1.089	0.014	77.321	0.926	***
F1	3	I felt that the health professional was open to questions during prenatal visits.	1.151	0.023	50.730	0.936	***	1.122	0.014	81.607	0.954	***
F1	4	I felt that the health professional cared about me.	1.114	0.025	44.134	0.936	***	1.096	0.014	79.152	0.932	***
F2	5	I felt that the health professional spent enough time discussing prenatal screening with me.	1.000			0.893		1.000			0.900	
F2	6	I felt that the health professional was open to questions about prenatal screening.	1.097	0.015	73.729	0.989	***	1.084	0.011	94.757	0.976	***
F2	8	I trust my health professional in matters related to prenatal screening.	0.754	0.030	25.399	0.673	***	0.688	0.029	23.527	0.619	***
F1	8	I trust my health professional in matters related to prenatal screening.	0.246	0.037	6.702	0.200	***	0.286	0.034	8.357	0.243	***

Note. *** = *p* < 0.001; B = unstandardized estimates; SE = standardized error; β = standardized estimates

**Fig. 3:**
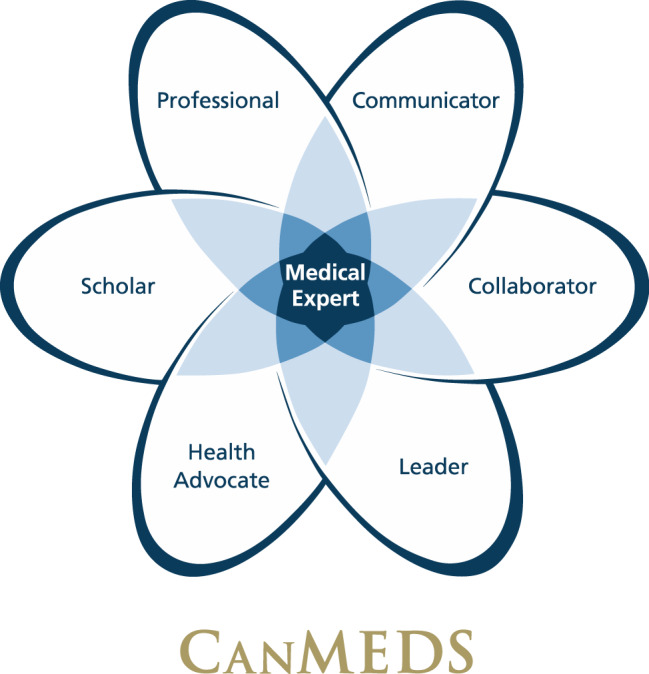
Royal College of Physicians and Surgeons of Canada’s CanMEDS framework [29]. Copyright © 2015 the Royal College of Physicians and Surgeons of Canada. https://www.royalcollege.ca/rcsite/canmeds/canmeds-framework-e. Reproduced with permission.

Furthermore, a domain expert confirmed that the two identified factors were aligned with two components of the principle framework used for physician education in Canada: The Royal College of Physicians and Surgeons of Canada’s CanMEDS framework (Fig. 3). This framework outlines the competencies physicians must have to meet the needs of their patients. Factor 1 was associated with the competency “Communication” and Factor 2 with “Professionalism.”

### Comparison of the two study arms

Data from 5913 pregnant women were included in the comparison, comprising 4111 participants in the first-tier (universal) cfDNA screening arm and 1802 in the standard care arm. Only participants with fully completed questionnaires and available socio-demographic data were analyzed. No significant differences were observed between the first-tier cfDNA screening arm and the standard care arm for either Factor 1 (Communication) or Factor 2 (Professionalism) (Table 8).

**Table 8:** Comparison of the PREM-PS scores by treatment arm using mixed models

	Intervention arm (cfDNA prenatal screening)			Control arm			Estimate difference (95% CI)
PREM-PS Score	N	Mean (SD)	Standardized mean	N	Mean (SD)	Standardized mean	
Factor 1	4111	−0.22 (24.89)	−0.009	1802	0.60 (24.66)	0.024	−0.75 (−2.11; 0.64)
Factor 2	4111	0.50 (30.88)	0.016	1802	−1.00 (30.94)	−0.032	1.48 (−0.30; 3.29)

Note. CI: Confidence interval; SD: Standard deviation

## Discussion

This study aimed to validate the PREM-PS questionnaire by evaluating its structural validity, internal consistency, and cross-cultural validity in both French- and English-speaking Canadian populations, while also comparing the experiences of participants randomized to either first-tier cfDNA screening or the traditional prenatal screening pathway. Key findings demonstrate that the PREM-PS effectively measures the experiences of pregnant women across both French and English-speaking populations. Also, it aligns with an established conceptual framework for medical education in Canada, the CanMEDS (Fig. 3). Results showed no difference when comparing the experiences of participants randomized to either first-tier cfDNA screening or the traditional prenatal screening pathway. These results lead us to make the following observations.

First, our findings indicate that the PREM-PS questionnaire measures two separate factors describing patients’ experiences, with good internal consistency across both the French and English versions, as demonstrated by Cronbach’s alpha. This means that this newly developed questionnaire can be used with confidence to collect patient-reported experience data in prenatal care settings across both language groups. The clinical and practical interpretation of PREM-PS scores is not straightforward, as it is limited by factors such as the absence of established benchmarks or threshold values; further research could investigate this aspect to enhance its applicability.

Second, Factor 1 was associated with “Communication” and Factor 2 with “Professionalism,” which align with components of the Royal College of Physicians and Surgeons of Canada’s CanMEDS framework (Fig. 3). The Communicator role describes the abilities related to a physician–patient encounter. Physicians enable patient-centered therapeutic communication by exploring the patient’s symptoms and by actively listening to the patient’s experience of their illness [29]. The Professional role reflects contemporary society’s expectations of physicians, which include clinical competence, a commitment to ongoing professional development, promotion of the public good, adherence to ethical standards and values such as integrity, honesty, altruism, humility, respect for diversity, and transparency with respect to potential conflicts of interest [29]. This two-factor structure echoes findings from previous research that identified similar relational dimensions in patient-reported experience measures. A Danish study aimed at developing a disease-specific PREM for patients with heart disease identified a nine-factor structure through EFA and CFA. Among these, *communication* and *information on disease and treatment* emerged as key components of the patient experience [30]. Although the questionnaires targeted different clinical populations, the conceptual overlap is notable. Their communication-related items—such as being listened to, being taken seriously, and having opportunities to ask questions—closely resemble items in our Communication factor (e.g., being treated with respect by the healthcare professional, healthcare professional showing care, healthcare professional being open to questions). Similarly, the Danish dimension *information on disease and treatment* shares conceptual ground with our Professionalism factor. While pregnancy is not a disease, patients in prenatal care also value being well-informed about procedures such as prenatal screening and being able to trust the information provided by healthcare professionals [31]. In the PREM-PS, the Professionalism-related items parallel components from the Danish instrument that assess patients’ perceptions of being informed about tests/examinations and their potential future health implications. The alignment between the factorial solution and the CanMEDS physician competency framework supports the relevance of the PREM-PS as a conceptually grounded instrument for assessing patient-reported experiences. Future research could investigate whether the PREM-PS can be adapted for use in other specialized healthcare settings while maintaining its connection to the CanMEDS framework, potentially contributing to a standardized approach to measuring patient-reported experiences across medical specialties.

Third, the comparison of patient experiences between the two randomized arms revealed no statistically significant differences for either the Professionalism or Communication factors. These findings suggest that patient-reported experiences of care were comparable across both arms, regardless of the screening method employed. While the retained CFA model showed some differences in factor loadings between the two language versions, each randomized group included participants who completed the questionnaire in both French and English. The similarity in factor loadings across the two language versions suggests that this limitation likely has minimal practical impact on the overall comparability of results. Our finding of a lack of significant difference in patient-reported experience between the two screening arms suggests that the quality of care was perceived similarly, regardless of the type of prenatal screening offered. This is consistent with the view that cfDNA prenatal screening does not negatively affect patients’ perception of their healthcare experience. In a UK-based study assessing the implementation of cfDNA screening within public maternity care [32] women were “overwhelmingly positive about their experience,” valuing the opportunity to have a test that was procedurally safe, accurate, simple to conduct, and which reduced the need for invasive testing.

Lastly, this bilingual study could be replicated in other settings where speakers of minority languages have been historically neglected by healthcare systems [33]. In order to advance equity in patient-centered measurement, further research should pursue cultural and linguistic comparisons of patient-reported experiences and make targeted efforts to mitigate cultural differences in responding to questionnaires as was manifested in our study [34].

This study has a few limitations. The high proportion of participants of European ancestry in the French-speaking group may limit the generalizability of the findings to other populations. However, within the French-speaking group, the percentage of participants of non-European descent was 19%, only slightly smaller than the overall Canadian percentage of 26% [35]. Also, ceiling effects were observed, with a substantial proportion of participants selecting the maximum response option for several items (Table 2). Future studies could consider modifying the response scale to reduce ceiling effects and better capture variability in patient experiences. Future research could focus on validating the PREM-PS in more diverse populations and exploring its application in other prenatal care settings. Additional psychometric testing such as hypotheses testing, responsiveness, and test-retest on a more diverse sample size could further verify the validity of the instrument.

## Conclusions

The PREM-PS is the first validated instrument specifically designed to measure patient-reported experiences in prenatal screening and demonstrates robust psychometric properties in both French- and English-speaking Canadian populations. This makes it a valuable and reliable instrument for assessing how women perceive the quality of care they receive, while also aligning with an established conceptual framework for medical education in Canada, the CanMEDS. The findings of this study suggest no differences in patient-reported experiences between the two study arms (first-tier cfDNA screening vs. standard care), indicating that both approaches provide comparable experiences. This validated instrument has the potential to guide improvements in prenatal care delivery and the evaluation of clinical practice.

## Data Availability

The datasets generated and/or analysed during the current study are not publicly available due to privacy considerations but are available from the corresponding author on reasonable request.

## Appendix 1

**Table 9:** Development of PREM-PS items

Item	Source (Adapted from PreMaPEQ/Newly developed)	Original PreMaPEQ item (if applicable)	Final English version	First French version	Final French version
		Response scale: − Not at all − To a small extent − To some extent − To a large extent − To a very large extent	Response scale: − Strongly disagree − Disagree − Neither agree nor disagree − Agree − Strongly agree	Response scale: − Pas du tout − Un peu - À un certain degré − Beaucoup - Tout à fait	Response scale: − Pas du tout d’accord - En désaccord − Ni d’accord, ni en désaccord − En accord - Tout à fait d’accord
1	Adapted from PreMaPEQ	Did the midwife treat you politely and with respect?	I was treated with politeness and respect by my health professional.	Avez-vous été traitée avec politesse et respect par la sage-femme, le médecin de famille ou l’obstétricien?	J’ai été traitée avec politesse et respect par le professionnel de la santé.
2	Adapted from PreMaPEQ	Did the midwife spend enough time at the visits?	I felt that the health professional spent enough time at the visits.	Votre sage-femme/médecin de famille/obstétricien a-t-il passé suffisamment de temps lors des visites?	J’ai trouvé que le professionnel de la santé a passé suffisamment de temps lors des visites.
3	Adapted from PreMaPEQ	Did you find that the midwife was open to your questions?	I felt that the health professional was open to questions during prenatal visits.	Avez-vous trouvé que votre sage-femme/médecin de famille/obstétricien était ouvert aux questions lors des visites prénatales?	J’ai trouvé que le professionnel de la santé était ouvert aux questions lors des visites prénatales.
4	Adapted from PreMaPEQ	Did you find that the midwife cared about you?	I felt that the health professional cared about me.	Avez-vous eu le sentiment que la sage-femme, le médecin de famille ou l’obstétricien se souciait de vous?	J’ai le sentiment que le professionnel de la santé se souciait de moi.
5	Newly developed	-	I felt that the health professional spent enough time discussing prenatal screening with me.	À votre avis, la sage-femme, le médecin de famille ou l’obstétricien a-t-il passé assez de temps à discuter de dépistage prénatal avec vous?	J’ai trouvé que le professionnel de la santé a passé assez de temps à discuter de dépistage prénatal avec moi.
6	Newly developed	-	I felt that the health professional was open to questions about prenatal screening.	Avez-vous trouvé que votre sage-femme/médecin de famille/obstétricien était ouvert aux questions?	J’ai trouvé que le professionnel de santé était ouvert aux questions relatives au dépistage prénatal.
7	Newly developed	-	I think the health professional influenced my decision to have screening or to not have screening.	À votre avis, est-ce que la sage-femme, le médecin de famille ou l’obstétricien a influencé votre décision de passer ou non un test de dépistage?	Je pense que le professionnel de la santé a influencé ma décision de passer ou non un test de dépistage.
8	Newly developed	-	I trust my health professional in matters related to prenatal screening.	Aviez-vous confiance dans les connaissances en matière de dépistage prénatal de votre sage-femme, votre médecin de famille ou votre obstétricien?	J’ai confiance en mon professionnel de la santé en matière de dépistage prénatal.
9	Newly developed	-	The health professional spent enough time discussing the results of my prenatal screening with me.	Est-ce que la sage-femme, le médecin de famille ou l’obstétricien a passé assez de temps à discuter des résultats de votre dépistage prénatal avec vous?	Le professionnel de la santé a passé assez de temps à discuter des résultats de mon dépistage prénatal avec moi.
10	Newly developed	-	My experience with prenatal screening was what I expected based on the information I received prior to screening.	Votre expérience en matière de dépistage prénatal correspond-t-elle aux informations que vous aviez reçues de la part de votre professionnel avant le dépistage prénatal?	Mon expérience en matière de dépistage prénatal correspond aux informations que j’avais avant le dépistage prénatal.

Note. Items adapted from PreMaPEQ were modified as follows: (1) format changed from question to statement; (2) “midwife” replaced with “health professional” to reflect the broader range of providers involved in prenatal screening; (3) response scale changed from a 5-point extent scale (“not at all” to “to a very large extent”) to a 5-point Likert agreement scale (“strongly disagree” to “strongly agree”). Cognitive interviewing led to refinement of the French response scale and modification of one item to avoid redundancy

## Appendix 2

**Table 10:** Descriptive statistics of participants by randomized arm and language of the questionnaire (a: French, b: English)

**a**			
**Characteristics**	**Overall** **n = 3260**	**Standard care** **n = 1013**	**cfDNA prenatal screening** **n = 2247**
Mean (SD) age (years)	31.02 (3.92)	30.87 (3.90)	31.08 (3.93)
Race/Ethnicity, n (%)			
Arab (Egypt, Iraq, Jordan, Lebanon)	110 (3.4)	36 (3.6)	74 (3.3)
East Asian (China, Japan, Korea, Taiwan)	34 (1.0)	11 (1.1)	23 (1.0)
West Asian (Turkey, Iran, Afghanistan)	13 (0.4)	4 (0.4)	9 (0.4)
South East Asian (Malaysia, Indonesia, Vietnam, Cambodia)	26 (0.8)	4 (0.4)	22 (1.0)
South Asian (Indian, Sri Lanka, Pakistan, Bangladesh)	8 (0.2)	3 (0.3)	5 (0.2)
Aboriginal (First Nations, Métis, Inuit)	3 (0.1)	2 (0.2)	1 (0.0)
White (European descent)	2657 (81.5)	831 (82.0)	1826 (81.3)
Latin American/Hispanic	75 (2.3)	23 (2.3)	52 (2.3)
Black (African descent)	92 (2.8)	23 (2.3)	69 (3.1)
Black (Caribbean descent)	92 (2.8)	28 (2.8)	64 (2.8)
Mixed ethnicity or other	149 (4.6)	48 (4.7)	101 (4.5)
Diabetes = Yes (%)	37 (1.1)	10 (1.0)	27 (1.2)
Hypertension = Yes (%)	48 (1.5)	13 (1.3)	35 (1.6)
Smoking = Yes (%)	120 (3.7)	47 (4.6)	73 (3.3)
Previous History of Pregnancy Preeclampsia = Yes (%)	100 (3.1)	32 (3.2)	68 (3.0)

**b**			
**Characteristics**	**Overall** **n = 2790**	**Standard care** **n = 824**	**cfDNA prenatal screening** **n = 1966**
Mean (SD) age (years)	33.43 (3.72)	33.58 (3.57)	33.37 (3.78)
Race/Ethnicity, n (%)			
Arab (Egypt, Iraq, Jordan, Lebanon)	29 (1.0)	6 (0.7)	23 (1.2)
East Asian (China, Japan, Korea, Taiwan)	677 (24.3)	193 (23.4)	484 (24.6)
West Asian (Turkey, Iran, Afghanistan)	35 (1.3)	10 (1.2)	25 (1.3)
South East Asian (Malaysia, Indonesia, Vietnam, Cambodia)	169 (6.1)	41 (5.0)	128 (6.5)
South Asian (Indian, Sri Lanka, Pakistan, Bangladesh)	198 (7.1)	46 (5.6)	152 (7.7)
Aboriginal (First Nations, Metis, Inuit)	6 (0.6)	1 (0.1)	5 (0.3)
White (European descent)	1282 (45.9)	416 (50.5)	866 (44.0)
Jewish	6 (0.2)	2 (0.2)	4 (0.2)
Latin American/Hispanic	163 (5.8)	48 (5.8)	115 (5.8)
Black (African descent)	20 (0.7)	7 (0.8)	13 (0.7)
Black (Caribbean descent)	7 (0.3)	0 (0.0)	7 (0.4)
Mixed ethnicity or other	198 (7.1)	54 (6.6)	144 (7.3)
Diabetes = Yes (%)	16 (0.6)	3 (0.4)	13 (0.7)
Hypertension = Yes (%)	16 (0.6)	5 (0.6)	11 (0.6)
Smoking = Yes (%)	15 (0.5)	2 (0.2)	13 (0.7)
Previous History of Pregnancy Preeclampsia = Yes (%)	37 (1.4)	12 (1.5)	25 (1.3)

## Abbreviations

cfDNA: Cell-free deoxyribonucleic acid
PREM: Patient-Reported Experience Measure
PreMaPEQ: Norwegian Pregnancy and Maternity care Patient’ Experience Questionnaire
KMO: Kaiser-Meyer-Olkin
EFA: Confirmatory Factor Analysis
CFA: Exploratory Factor Analysis
WLSMV: Weighted Least Squares Mean and Variance-adjusted
CFI: Comparative Fit Index
RMSEA: Root Mean Square Error of Approximation
MIs: Modification Indices
